# Genetic variation and risks of introgression in the wild *Coffea arabica* gene pool in south-western Ethiopian montane rainforests

**DOI:** 10.1111/j.1752-4571.2012.00285.x

**Published:** 2012-07-18

**Authors:** Raf Aerts, Gezahegn Berecha, Pieter Gijbels, Kitessa Hundera, Sabine Glabeke, Katrien Vandepitte, Bart Muys, Isabel Roldán-Ruiz, Olivier Honnay

**Affiliations:** 1 Division Forest, Nature and Landscape, University of LeuvenLeuven, Belgium; 2 Department of Horticulture and Plant Science, Jimma UniversityJimma, Ethiopia; 3 Division of Ecology, Evolution and Biodiversity Conservation, University of LeuvenLeuven, Belgium; 4 Department of Biology, Jimma UniversityJimma, Ethiopia; 5 Unit Plant, Institute for Agricultural and Fisheries Research ILVOMelle, Belgium

**Keywords:** admixture, Afromontane rainforest, coffee, crop wild relative, ecosystem services, genetic erosion

## Abstract

The montane rainforests of SW Ethiopia are the primary centre of diversity of *Coffea arabica* and the origin of all Arabica coffee cultivated worldwide. This wild gene pool is potentially threatened by forest fragmentation and degradation, and by introgressive hybridization with locally improved coffee varieties. We genotyped 703 coffee shrubs from unmanaged and managed coffee populations, using 24 microsatellite loci. Additionally, we genotyped 90 individuals representing 23 Ethiopian cultivars resistant to coffee berry disease (CBD). We determined population genetic diversity, genetic structure, and admixture of cultivar alleles in the *in situ* gene pool. We found strong genetic differentiation between managed and unmanaged coffee populations, but without significant differences in within-population genetic diversity. The widespread planting of coffee seedlings including CBD-resistant cultivars most likely offsets losses of genetic variation attributable to genetic drift and inbreeding. Mixing cultivars with original coffee genotypes, however, leaves ample opportunity for hybridization and replacement of the original coffee gene pool, which already shows signs of admixture. *In situ* conservation of the wild gene pool of *C. arabica* must therefore focus on limiting coffee production in the remaining wild populations, as intensification threatens the genetic integrity of the gene pool by exposing wild genotypes to cultivars.

## Introduction

To improve quality, achieve higher yields, or create pest and disease-resistant or stress-tolerant varieties of crops, plant breeders often utilize crop wild relatives (CWRs) (Hoisington et al. 1999; Heywood et al. 2007; Lashermes et al. 2011). CWRs are progenitors of crops and wild plant taxa that have relatively close genetic relationships to crops, but that are not domesticated themselves (Meilleur and Hodgkin 2010; Maxted et al. 2006). CWRs may possess desirable characteristics that can be used to improve existing crops (Gur and Zamir 2004; Fernie et al. 2006; Maxted et al. 2007; Takeda and Matsuoka 2008). In particular, in the light of global climatic change, sustained agricultural production may increasingly rely on the genetic enhancement of crops using the diverse germplasm of CWRs (Heywood et al. 2007; Tester and Langridge 2010; Foley et al. 2011; Ford-Lloyd et al. 2011), and for that reason, the *in situ* conservation of the genetic diversity of CWRs is an important but often undervalued challenge (Mercer and Perales 2010; Honnay et al. 2012).

Arabica coffee is one of the world's most valuable agricultural commodities, accounting for two-thirds of the global coffee market (Labouisse et al. 2008). Despite the currently wide geographical range of arabica coffee cultivation, the number of cultivars used is very small: mainly *Coffea arabica* var. *typica*, *C. arabica* var. *bourbon* and hybrids of the two (Labouisse et al. 2008). The narrow genetic base of those cultivars (Anthony et al. 2002) has resulted in a crop with homogenous agronomic behaviour (Lashermes et al. 2009), but also with a high susceptibility to biotic and climatic hazards (Labouisse et al. 2008; Jaramillo et al. 2011), and a low adaptability in response to environmental changes or changing market demands.

The closest wild relative of cultivated arabica coffee is wild *Coffea arabica*, which has its origin and centre of diversity in south-western Ethiopia (Anthony et al. 2001, 2002). Wild arabica coffee is a unique potential source of genetic diversity for selection and breeding of enhanced arabica cultivars, including varieties with low caffeine content, increased yields, or increased resistance to pests and pathogens such as coffee berry disease (CBD, caused by *Colletotrichum kahawae*), coffee rust (caused by *Hemileia vastatrix*), *Meloidogyne* root nematodes and the coffee berry borer (*Hypothenemus hampei*) (Hein and Gatzweiler 2006; Silvestrini et al. 2007; Dessalegn et al. 2008; Boisseau et al. 2009). Despite its importance for the global coffee industry and for the livelihood of rural communities depending on coffee cultivation, the status of the wild gene pool of arabica coffee is largely unknown and potentially threatened (Labouisse et al. 2008), a fate shared with many other CWRs in the world (Heywood et al. 2007).

Two major anthropogenic processes may potentially threaten the diversity and integrity of the gene pool of wild *Coffea arabica*: (i) the fragmentation and intensive management of the natural Afromontane rainforests, and (ii) the large scale introduction of improved coffee varieties in natural coffee stands. First, as elsewhere in the tropics (Ahrends et al. 2010; DeFries et al. 2010), forest conversion to agriculture and other land uses related to urban population growth have resulted in the fragmentation of the Ethiopian montane forest (Gole et al. 2008). Furthermore, traditional forest coffee production practices in Ethiopia also alter forest structure and plant communities (Schmitt et al. 2009; Aerts et al. 2011). The intensity of management varies between so-called forest coffee (FC) systems, which undergo little or no intervention, and semi-forest coffee (SFC) systems, in which herbs, shrubs (other than coffee) and emerging tree seedlings in the understory are removed annually, the upper canopy is selectively thinned and coffee saplings are locally planted (Senbeta and Denich 2006; Schmitt et al. 2009; Aerts et al. 2011). Both forest fragmentation and forest degradation can have a negative impact on the genetic diversity of forest plant species through increased genetic drift, reduced gene flow, and alteration of mating patterns resulting in increased inbreeding (Young et al. 1996; Honnay et al. 2005; Eckert et al. 2010). Second, the widespread planting, since the 1970s, of a restricted set of locally improved coffee varieties, mainly genotypes resistant to coffee berry disease (CBD), in the forest and its surroundings may result in the replacement of a part of the wild gene pool with a small number of domesticated alleles (Ellstrand et al. 1999; Becker et al. 2006; Hooftman et al. 2007). This can result in loss of genetic variation from the original gene pool and may even have negative fitness consequences for the original populations (Ellstrand 2003).

The general aim of this study was to provide the first thorough assessment of population genetic diversity within the wild gene pool of *Coffea arabica* in its centre of origin, the south-western Ethiopian montane rainforests. We addressed the following specific questions: (i) Is there genetic erosion of the wild arabica gene pool in fragmented forests managed for coffee production, compared to continuous nonmanaged forests? (ii) Is the introduction of CBD-resistant genotypes posing a threat to the integrity of the wild arabica gene pool? The answers to these questions should help the *in situ* conservation of arabica coffee genetic resources.

## Materials and methods

### Study species

*Coffea arabica* L. (family Rubiaceae) is the only *Coffea* species occurring in Ethiopia and is geographically isolated from all other species in the genus (Silvestrini et al. 2007). It is a naturally occurring understory shrub of the Afromontane rainforest, a type of moist evergreen montane rainforest found in the south-western highlands between 1500 and 2600 m, with an annual rainfall between 700 and 1500 mm (Friis 1992). The canopy of the Afromontane rainforest typically consists of a mixture of broad-leaved species 10-30 m tall with emergent trees that may reach a height of 30–40 m (Demissew et al. 2004). Wild coffee generally occurs between 1500 and 1900 m, but cultivated plants are found over a much wider range, between 1000 and 2800 m (Hedberg et al. 2003; Gole et al. 2008). Flowering is induced by rains, with a short annual period of synchronous flowering usually in January. The species is self-compatible and mainly insect-pollinated, in Ethiopia typically by bees, which are attracted to the nectar (Fichtl and Admasu 1994). *Coffea arabica* fruits take about 1 year to reach maturity and are dispersed by birds, bats, monkeys, rodents and humans. Its population density varies by forest management intensity, with on average 3900 individuals (≥0.5 m in height and dbh ≥2 cm) ha^−1^ in the FC system compared to 18 500 individuals ha^−1^ in the SFC system (Schmitt et al. 2009). Within the genus, it is the only allotetraploid (2*n* = 4*x* = 44), formed by relatively recent natural hybridization between *C. canophora* and *C. eugenioides* (Lashermes et al. 1999). The recent origin and self-fertilization of *C. arabica* probably contribute to its relatively low genetic diversity compared to diploid *Coffea* species (Lashermes et al. 2000).

### Sample collection and DNA extraction

**Figure 1:**
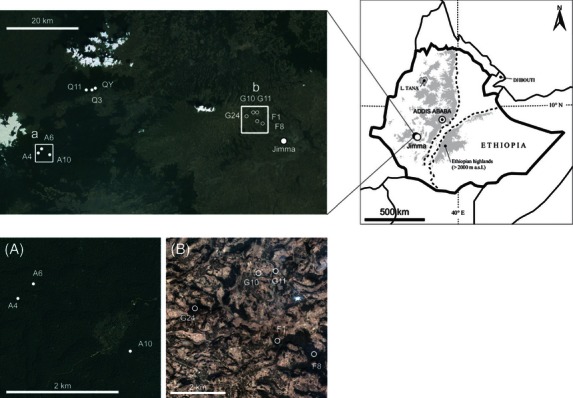
Afromontane rainforests in Southwest-Ethiopia and sampled *Coffea arabica* populations: forest coffee (closed circles) and semi-forest coffee (open circles). Insets show detail of the forest coffee (A) and of the semi-forest coffee landscape (B). Satellite imagery © 2012 DigitalGlobe, GeoEye and Cnes/Spot Image, via Google Earth.

Coffee leaf samples were collected in eleven coffee stands in montane rainforest in the Jimma zone of Oromia region in SW Ethiopia (Table 1, Fig. 1). Six stands were located in the remote Gera sector of the Belete–Gera National Forest Priority Area (NFPA) and were classified as FC. These stands showed no or only few signs of forest management. Five other stands were located in forest fragments (1–20 ha in size), which are managed as SFC since the 1970s and which are located in the coffee-producing agricultural landscape east of the NFPA. All these forest fragments showed clear evidence of tree thinning, understory removal and locally, of coffee planting activities (including CBD-resistant cultivars), as revealed, for instance, by the high density and regular spacing of coffee plants (Aerts et al. 2011).

**Table 1:** Location, sample size, molecular variance (*MV*), expected heterozygosity corrected for sample size *H*_*E,C*_ and population mean STRUCTURE cluster membership coefficients *Q* for eleven *Coffea arabica* stands and 23 cultivars in SW Ethiopia

Stand	Lat (N)	Long (E)	Elev (m)	*N*	*MV*	*H*_*E,C*_	*Q*		

							I	II	SE
*Forest coffee*									
Afalo (A10)	7.6307	36.2241	1825	62	23.27	0.509	0.27	0.73	0.042
Afalo (A6)	7.6404	36.2092	1889	73	13.77	0.565	0.56	0.44	0.112
Afalo (A4)	7.6395	36.2067	1987	73	20.90	0.561	0.42	0.58	0.042
Qacha (Q11)	7.7868	36.3238	2108	63	15.82	0.600	0.67	0.33	0.086
Qacha (Q3)	7.7817	36.3313	1920	88	30.41	0.574	0.46	0.54	0.107
Qacha (QY)	7.7865	36.3432	1926	24	21.28	0.623	0.80	0.20	0.043
*Semi-forest coffee*									
Fetche (F1)	7.7144	36.7482	2085	60	25.06	0.552	0.78	0.22	0.003
Fetche (F8)	7.7106	36.7617	1908	61	14.80	0.554	0.81	0.19	0.031
Garuke (G10)	7.7368	36.7420	2025	57	17.63	0.560	0.69	0.31	0.069
Garuke (G11)	7.7373	36.7477	2040	82	23.99	0.547	0.57	0.43	0.138
Garuke (G24)	7.7256	36.7227	2062	60	20.01	0.500	0.62	0.38	0.144
*Cultivars*				90	23.25	0.621	0.58	0.42	0.053

In each stand, we established rectangular plots, containing approximately 65 coffee shrubs. Plots were at least 20 m away from the edge in SFC forest fragments. Because of the difference in coffee density between forest stands, these plots varied in size between 12 and 225 m^2^. All shrubs within a plot were sampled for young leaf material, totalling 703 samples across 11 plots. In one forest stand (QY, Table 1), coffee density was very low and we sampled only 24 individuals. In two other stands (G11 and Q3), coffee density was higher than estimated when establishing the plots and we sampled 82 and 88 individuals, respectively. The coffee plants within a plot are further referred to as a population. This set was complemented with leaf samples of 90 individuals representing 23 different CBD-resistant varieties (741, 744, 7440, 7454, 7487, 74110, 74112, 74140, 74148, 74158, 74165, 754, 75227, Ababuna, Bunawashi, Dessu, Gawe, Gesha, Melko-CH2, Me'oftu, Merdahereka, Wushwush and Yachi; hereafter called ‘cultivars’), which were locally developed by the Ethiopian Institute of Agricultural Research from genotypes collected throughout the Ethiopian montane rainforests and which have been released between the late 1970s and 1990s.

Leaf material was dried on silica gel. Before DNA extraction, leaves were freeze-dried for 48 h and homogenized with a mill (Mixel Mill MM 200; Retsch®, Haan, Germany). Genomic DNA was extracted from 20 mg homogenized leaf material using the NucleoSpin® Plant II kit (Machery-Nagel, Düren, Germany), with slight modifications of the standard CTAB protocol (we increased the incubation time during cell lysis to 60 min at 65°C and used a two-step elution procedure incubated at 70°C for optimal recovery of bound nucleic acids).

### Simple sequence repeats genotyping

Twenty-four simple sequence repeats (SSRs, microsatellites) were amplified in six multiplex PCRs (Table S1) using a GeneAmp^*®*^ PCR System 9700 thermal cycler (Applied Biosystems^*®*^, CA, USA) and a total sample volume of 10 μL containing 5 μL Qiagen^*®*^ Multiplex PCR Master Mix (Qiagen, Valencia, CA, USA), 2 μL sample/template DNA and 0.2 μL of each primer (reverse and forward, 10 μm) in the multiplex combination complemented with RNase-free Milli-Q water. The multiplexes had equal thermocycling profiles with an initial *Taq* DNA polymerase heat-activation step at 95°C for 15 min; 25 cycles of 30 s at 94°C (denaturation step), 90 s at 57°C (annealing step) and 60 s at 72°C (extension step); and a final extension step of 30 min at 60°C. Then, 1 μL of the PCR was added to a solution of 8.8 μL formamide and 0.2 μL of the Applied Biosystems GeneScan™ 500 LIZ^*®*^ size standard. Sized fragments were scored using GeneMapper^*®*^ v4.0 (Applied Biosystems).

### Genetic data analysis

The allotetraploid nature of the *Coffea arabica* genome limits the flexibility of the data analysis. We adopted two parallel approaches, one based on the codominantly scored data and allowing allele copy number ambiguity, and a second based on the scoring of each individual allele as present or absent, resulting in a dominantly scored data set comparable to the output of an amplified fragment length polymorphism (AFLP) marker approach. We used the R package polysat (Clark and Jasieniuk 2011) as a central data handling facility, that is, for importing the SSR data from the GeneMapper^*®*^ software and converting the data. To assess the resolution of the microsatellite marker set we discriminated distinct multilocus genotypes (MLGs).

### Genetic diversity and population differentiation

Population genetic diversity was quantified using the expected heterozygosity corrected for sample size (*H*_*E,C*_), and the molecular variance (*MV*) based on the within-population sum of squares (*SSWP*) and calculated as *SSWP*×(*n* − 1)^−1^. Among-population genetic differentiation (*Φ*_PT_) was calculated based on Euclidian genetic distances (Huff et al. 1993). *H*_*E,C*_ is based on the tetraploid data set and was calculated in ATETRA 1.2.a (Van Puyvelde et al. 2010), whereas *MV* and *Φ*_PT_ resulted from a hierarchical analysis of molecular variance (amova) approach on the dominantly scored data set as performed in GenAlEx 6.41 (Peakall and Smouse 2006). For the amova, we used the coffee production system (FC vs SFC) as the regional grouping variable. Genetic differentiation was further assessed using principal coordinates analyses (PCoA) calculated in GenAlEx on the pairwise *Φ*_PT_ matrix. Effects of coffee forest management intensity (FC vs SFC) on population genetic diversity were analysed using Wilcoxon–Mann–Whitney *U*-tests. To test whether the mean genetic diversities recorded in FC and SFC populations differed from the diversity in the cultivar population, we used one-sample *t*-tests. Finally, also the average pairwise genetic differentiation (*Φ*_PT_) between populations in FC stands was compared with the differentiation between populations in SFC stands, using a Wilcoxon–Mann–Whitney *U* test. Statistical analyses were performed in SPSS 15.0 (SPSS Inc., Chicago, IL, USA).

### Bayesian analysis of population structure

To investigate the presence of alleles from the cultivar gene pool across the coffee populations, genetic structure was assessed using Bayesian clustering analysis implemented in structure 2.3.3 (Pritchard et al. 2000, 2010; Falush et al. 2007). structure was run five times at *K* = 1–9 applying 10 000 burn-in cycles and 50 000 Markov Chain Monte Carlo (MCMC) iterations. Correlated allele frequencies and admixture were assumed. The degree of admixture was inferred from the data using an initial value of *α* = 1.0 and a maximum of 10. The value of *K* that best fitted our data was selected using the estimated log probability of data Pr(*X*|*K*) and the derived *ΔK* statistic (Evanno et al. 2005). We used clumpp 1.1.2 (Jakobsson and Rosenberg 2007) to match the five structure solutions and calculate average ancestry estimates, given as estimated membership coefficients *Q* for each individual and each population, in each of the *K* clusters.

### Analysis of genome-wide admixture

We assessed genome-wide admixture of alleles from the cultivar gene pool into the FC and SFC populations using a hybrid index or admixture coefficient (Gompert and Buerkle 2009), as calculated in the R package Introgress (Gompert and Buerkle 2010). The SSR data of the populations A10 and Q3 and of the cultivar population were used as wild and cultivar parental data, respectively, and the SSR data of the remaining FC and all SFC populations were entered as potentially admixed individuals. For this analysis, A10 and Q3 were selected as pure wild populations, because these were the only populations where the owners guaranteed that they had not planted any cultivars, and thus, where the introduction of coffee plants had not taken place. The est.h function of Introgress was applied to calculate a maximum likelihood hybrid index estimate *h* for each potentially admixed individual (Gompert and Buerkle 2010). We compared hybrid index means of FC and SFC using the independent-samples *t*-test.

## Results

### Genetic diversity and differentiation

**Figure 2:**
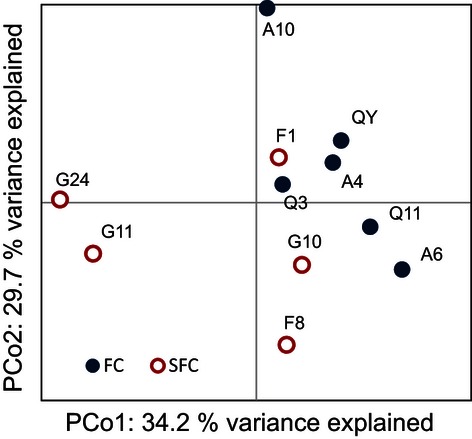
Principal coordinates (PCo) plot based on *Φ*_PT_ calculated with 24 SSR markers for *Coffea arabica*, demonstrating population genetic differentiation between forest coffee (closed circles) and semi-forest coffee systems (open circles).

The 24 SSRs in the six multiplex combinations yielded a total of 159 alleles. The number of alleles ranged from 2 to 19 per locus. Only eleven individuals were assigned to four nonunique MLGs across populations. The expected heterozygosity corrected for sample size (*H*_*E,C*_) and the molecular variance (*MV*) did not differ significantly between FC (*H*_*E,C*_ = 0.571, SE 0.016; *MV* = 20.91, SE 2.40) and SFC populations (*H*_*E,C*_ = 0.552, SE 0.014; *MV* = 20.30, SE 1.92) (*H*_*E,C*_: *U* = 10, *P =* 0.429; *MV*: *U* = 15, *P* = 1.000). The sample of CBD-resistant varieties was genetically more diverse (*H*_*E,C*_ = 0.621; *MV =* 23.25) than both the FC and SFC populations, but this difference was only significant for *H*_*E,C*_ (*t*_*10*_ = −5.90, *P* < 0.001). Overall among-population genetic differentiation was high (*Φ*_PT_ = 0.186, *P* < 0.001), with a genetic differentiation of 0.033 (*Φ*_*RT*_) between production systems (Table 2). Genetic differentiation among populations was significantly higher for the SFC (*Φ*_PT_ = 0.176, SE 0.018) than for the FC (*Φ*_PT_ = 0.131, SE 0.014) populations (*U* = 38, *P* = 0.040). Populations from FC and SFC clustered at opposite ends in the PCoA with some overlap among clusters (e.g. F1, Q3) (Fig. 2).

**Table 2:** Hierarchical analysis of molecular variance of 159 alleles at 24 microsatellite loci for 703 *Coffea arabica* individuals distributed in 11 stands and two coffee production systems, forest coffee (FC) and semi-forest coffee (SFC), in SW Ethiopia

Source	*df*	*SS*	*MS*	*EV*	*%MV*	*Φ*-statistic		*P*
Among systems (FC-SFC)	1	586.0	586.0	0.85	3.27	*Φ*_*RT*_	0.033	0.001
Among populations (stands)	9	2452.7	272.5	3.97	15.36	*Φ*_*PR*_	0.159	0.001
Within populations (stands)	696	14623.5	21.0	21.01	81.37	*Φ*_PT_	0.186	0.001
Total	706	17662.2		25.82				

For each source of variation the following is given: the number of degrees of freedom (*df*), the sum of squared difference to the mean (*SS*) and the mean sum of squares (*MS*), the estimated variance (*EV*), the percentage of total molecular variance (*%MV*), the *Φ*-statistic and the associated probability.

### Genetic structure and admixture

**Figure 3:**
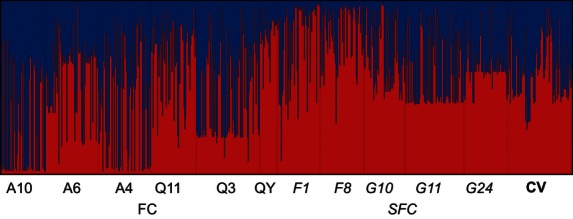
Population structure of *Coffea arabica* based on structure analysis of 24 SSR markers for forest coffee (*N =* 383, six populations), semi-forest coffee (*N* = 320, five populations) and cultivar (*N* = 90) samples for *K* = 2 clusters (Fig. S1). Individuals are represented by columns, with colours showing the average proportion (*R* = 5 runs) of their genome assigned to the different clusters, demonstrating the prevalence of a genotype associated to the coffee berry disease (CBD)-resistant gene pool in the semi-forest coffee (SFC) populations.

The log probability of data increased with increasing *K* but was less pronounced when *K* > 2. This, together with the fact that the *ΔK* statistic reached its maximum at *K* = 2 (Fig. S1), suggested the existence of two clusters. The genotypes assigned to cluster I were by far the most widespread and dominated five SFC populations and two FC populations. Genotypes assigned to cluster II were dominant in the remote FC population A10. One SFC population (G11), three FC populations (A6, A4, Q3) and the cultivars had comparable proportions of individuals assigned to either cluster I or II, as well as individuals with an admixed genotype, that is, assigned to both genetic clusters with comparable probabilities (Table 1; Fig. 3).

**Figure 4:**
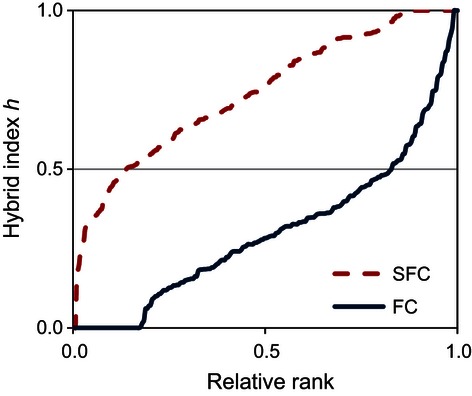
Maximum likelihood hybrid index estimates *h* for 320 *Coffea arabica* individuals from five semi-forest coffee populations and for 233 *Coffea arabica* individuals from four forest coffee populations in SW Ethiopia. The *h* index or admixture coefficient, based on frequencies for 159 alleles, a parental population of *N* = 150 wild individuals (from populations A10 and Q3) and a parental population of *N =* 90 specimens from 23 coffee berry disease (CBD)-resistant varieties, gives the fraction of the genome shared with the cultivated varieties for each individual.

The admixture coefficients or hybrid indices varied between 0 (pure ‘wild’) and 1 (pure ‘cultivar’). In the SFC populations, hybrid indices were predominantly high, with *h* > 0.50 for 86.3% of the samples. In the FC populations, hybrid indices were low, with *h* < 0.50 for 82.4% of the samples (Fig. 4). The mean hybrid index was significantly higher in the SFC (mean *h*_SFC_ = 0.74, SE 0.012; *h*_*FC*_ = 0.30; SE 0.015) (*t*_*551*_ = 22.4, *P* < 0.001). These results indicate that alleles from the cultivar gene pool are more prevalent in the SFC than in the FC populations, and that most individuals in the SFC populations have more alleles from the cultivar gene pool than alleles from the wild gene pool in their genome.

## Discussion

### Genetic diversity and cryptic genetic erosion

Although SFC management is associated with major changes in forest structure and shrub and canopy species composition (Schmitt et al. 2009; Aerts et al. 2011), it had no negative impact on coffee genetic diversity within populations (*H*_*E,C*_, *MV*). This was surprising because negative effects on effective population size and genetic diversity of *C. arabica* populations could have been expected. Changes in forest microclimate and in availability of nesting sites for insect pollinators, for instance, may have negatively affected pollinator diversity (Klein et al. 2008), reducing cross-pollination (Klein et al. 2003), increasing selfing and inbreeding (Eckert et al. 2010), and reducing progeny vigour and effective population size (Honnay and Jacquemyn 2007). As SFC systems are already managed as such for some generations (∼40 years), it is unlikely that expected genetic changes still have to become apparent. More plausible, and in agreement with our other results, is that farmers have been introducing new genotypes from elsewhere, both CBD-resistant genotypes provided by local authorities and wild genotypes from neighbouring sites, compensating for the eventual loss of genetic variation through genetic drift and inbreeding.

The introduction of genetically diverse coffee genotypes from elsewhere into SFC populations by coffee farmers most likely represents a farmer-mediated evolutionary force. This is supported by the higher genetic differentiation (*Φ*_PT_) among SFC populations than among FC populations. The conspicuous presence of alleles from the pool of introduced CBD-resistant genotypes in almost all SFC populations (high *h*, Fig. 4) also suggests that genetic diversity in the original coffee populations is being displaced, which is a form of cryptic genetic erosion (see Fig. S2). The local introduction of CBD-resistant genotypes in the SFC area since the 1970s probably facilitated later hybridization through gene flow by pollen (Papa and Gepts 2003). We can, however, not exclude that regional differences contributed to the higher genetic differentiation within the SFC region but we expect that their effect is limited. First, given that FC populations have been sampled over a much larger area (25 × 25 km^2^, Fig. 1) than the SFC populations (5 × 5 km^2^), this higher genetic differentiation is unlikely to be caused through isolation-by-distance or by greater environmental variation among SFC populations. Second, gene flow through pollen or seed between SFC populations may have been impeded due to the more fragmented nature of the agricultural landscape near Jimma town, increasing the among-population genetic differentiation. If the latter was the case, however, we expect genetic differentiation to be paralleled by loss of genetic diversity through genetic drift, which was not observed in the SFC populations.

### Introduction of alleles from CBD-resistant varieties

Gene flow from domesticated crops to wild relatives poses an important potential threat to CWRs, as repeated occurrences of hybridization may lead to the loss of the genetic integrity of the wild species (introgression), which becomes assimilated into the cultivar. This process has already increased the risk of extinction of the wild relatives of two of the world's 13 most important crops, rice (*Ozyra sativa*) and cotton (*Gossypium hirsutum*) (Ellstrand et al. 1999). Evidence of introgression from modern hybrid crop varieties into wild populations and populations of locally domesticated landraces is rapidly emerging (e.g. Sørensen et al. 2007; Bitocchi et al. 2009; Arnaud et al. 2010; Arrigo et al. 2011; Kwit et al. 2011), warning ecologists and plant breeders about the latent extinction of wild relative populations. The high genetic variation within the group of introduced CBD-resistant genotypes in this study (Table 1) strongly suggests that the improved varieties that have been released in Ethiopia did not yet undergo an extreme process of breeding and selection, and that the process of domestication so far was focussed on the capture and multiplication of genotypes with desirable traits. So far, coffee breeding in Ethiopia has been less intensive than other crops such as maize, which underwent a rigorous process of breeding and selection, yielding elite breeding pools with very little of the genetic diversity found in the maize wild relatives (Ortiz et al. 2010). In general, genetic domestication bottlenecks seem to be more limited in perennial fruit crops than in annual crops (Miller and Gross 2011), although commercial coffee cultivars grown in central America or Asia show important losses of genetic diversity (Lashermes et al. 1996; López-Gartner et al. 2009).

Our results show the striking presence of alleles from the CBD-resistant gene pool in all SFC populations, and to a much lesser extent in some FC populations. This suggests the possibility that introgression from recently introduced cultivars may be common in wild populations. Although the process of introgression may be slower in long-lived or clonal plants, such as coffee, postzygotic barriers to hybridization may be weaker than in domesticated annuals such as cereals, where hybrid seedlings have been shown to be maladapted to wild environments (McKey et al. [Bibr b54]). The CBD-resistant cultivars were released starting in the 1970s, providing ample opportunities for hybridization, given that the generation time for coffee is ∼3 years. Hybridization between cultivars and wild individuals has been reported in a number of long-lived perennials, such as grapevine (*Vitis vinifera*, De Andrés et al. 2012), wild almond (*Prunus orientalis*, Delplancke et al. 2012) and wild apple (*Malus sylvestris*, Coart et al. 2003). However, we cannot rule out that the presence of these alleles in wild populations may simply reflect the shared ancestry between introduced and wild genotypes. CBD-resistant genotypes are derived from FC trees from different parts of Ethiopia, and at least some of the alleles shared between the cultivar gene pool and the SFC are expected to be identical by descent. Further research should focus in depth on gene flow between wild coffee and introduced cultivars, for example through parentage analysis (De Andrés et al. 2012).

## Conclusions

Our results clearly show that SFC populations are genetically more similar to the pool of introduced CBD-resistant genotypes than FC populations, but that SFC populations are not less diverse than FC populations. These patterns can be explained by the anthropogenic introduction of genotypes (both wild genotypes and CBD-resistant cultivars) in SFC populations. Although we cannot provide direct evidence for hybridization and introgression, the practice of large scale planting of CBD-resistant genotypes by local farmers in SFC systems, intimately mixed with original coffee genotypes, offers ample opportunity for the exchange of alleles and the replacement of the original coffee gene pool.

Only the few remaining large forests with a FC cultivation system seem to be safe from the introduction of CBD-resistant cultivars so far. To ensure the *in situ* conservation of arabica coffee genetic resources in Ethiopia, we recommend to: (i) avoid establishing plantations with foreign coffee cultivars in the centre of origin of *C. arabica*; (ii) allow only the use of Ethiopian cultivars that did not undergo a very strict process of selection in the SFC systems; and (iii) protect a sufficiently large area of low-intensity FC systems. The latter can probably only be realized by establishing buffer zones of SFC surrounding more strict reserves of FC in the last remaining large forest blocks in the region.

## Data archiving statement

Data for this study are available at Dryad. DOI: 10.5061/dryad.37r9p.

## Supporting Information

**Figure S1.** *ΔK* statistic for detection of the true number of groups *K** or the value of *K* that best fit the data.

**Figure S2.** Relative frequency distribution of alleles for *Coffea arabica* microsattelite EU597612 (samples with missing values for this SSR were omitted), illustrating cryptic genetic erosion in the *in situ* arabica gene pool. The same four alleles are present in forest coffee (FC) and semi-forest coffee (SFC), but within the SFC two of these four alleles are rare. The *φ*-statistic and probability *P* show a significant association of two alleles to FC and one to SFC.

**Table S1.** Multiplex panels used for SSR genotyping of *Coffea arabica*.
