# Supplementary material for: Genetic variation and risks of introgression in the wild Coffea arabica gene pool in south-western Ethiopian montane rainforests
Source: Evol Appl. 2012 Jul 18;6(2):243–52. doi: 10.1111/j.1752-4571.2012.00285.x (PMC3689350; doi:10.1111/j.1752-4571.2012.00285.x)
Supplement: Supplementary file 1 [file eva0006-0243-SD1.doc]

**Supporting Information**


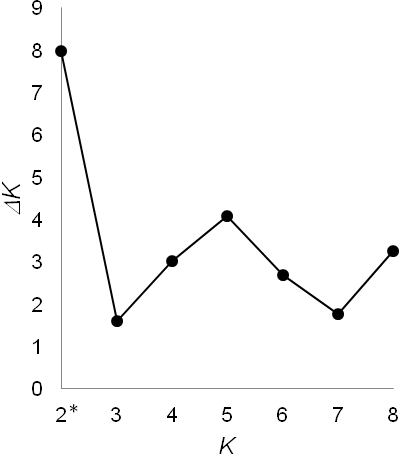


**Fig. S1** *ΔK* statistic for detection of the true number of groups *K** or the value of *K* that best fit the data


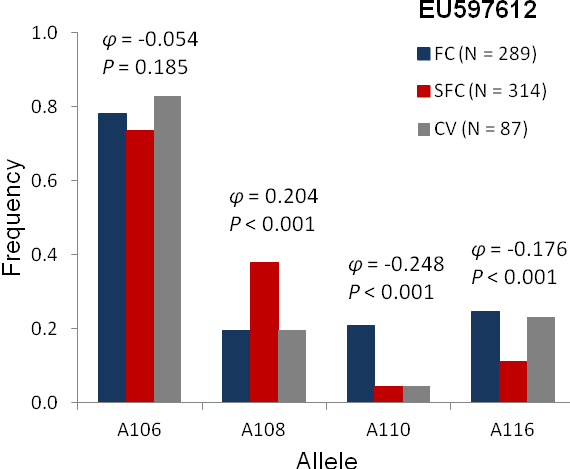


**Fig. S2** Relative frequency distribution of alleles for *Coffea arabica* microsattelite EU597612 (samples with missing values for this SSR were omitted), illustrating cryptic genetic erosion in the *in situ* arabica gene pool. The same four alleles are present in FC and SFC, but within the SFC two of these four alleles are rare. The *φ*-statistic and probability *P* show a significant association of two alleles to FC and one to SFC.

| **Table S1** Multiplex panels used for SSR genotyping of *Coffea arabica* | | | | | | |
| --- | --- | --- | --- | --- | --- | --- |
| Multiplex  panel |  | Genbank Accession^1^ | Dye^2^ |  | PCR primer sequences (5’-3’)  (F: forward; R: reverse) |  |
| A |  | AJ250253 | FAM |  | F: CTTGTTTGAGTCTGTCGCTG  R: TTTCCCTCCCAATGTCTGTA |  |
|  |  | AJ250254 | FAM |  | F: GGCTCGAGATATCTGTTTAG  R: TTTAATGGGCATAGGGTCC |  |
|  |  | AJ308742 | VIC |  | F: ggcttcttgggtgtctgtgt  R: ccattggctttgtatttctgg |  |
|  |  | AJ250255 | NED |  | F: CCCTCCCTGCCAGAAGAAGC  R: AACCACCGTCCTTTTCCTCG |  |
|  |  | AJ250256 | PET |  | F: AGGAGGGAGGTGTGGGTGAAG  R: AGGGGAGTGGATAAGAAGG |  |
| B |  | EU526567 | FAM |  | F: CCGACTTGGACTGATGCGAAATTGA  R: AAAGCAAAAAACCAGAAAACACGAAGA |  |
|  |  | EU526570 | VIC |  | F: CCCCTCCTCCTCCTACTAGATGGTGGT  R: GGTCCAGGGTCCATCCATTCTTGA |  |
|  |  | EU526586 | VIC |  | F: TGGGTCAAGGATCCGTGTAAGAAAGA  R: CCCTCACCAGTTCCCGATGTCAG |  |
|  |  | AJ308754 | NED |  | F: TACAAGGGGAGTGGATAAGA  R: GTTTGTAGGAGGAAGGTGTG |  |
|  |  | EU526558 | PET |  | F: CGCGCTTGCTCCCTCTGTCTCT  R: TGGGGGAGGGGCGGTGTT |  |
| C |  | EU597609 | FAM |  | F: AGCAACTTCGCCAGTCATTA  R: GCGGGTCTTATTCAACGTATAC |  |
|  |  | EU597604 | VIC |  | F: CCATTCTAACCAAACCTGTCC  R: CTCAAACACTTGGGTGTGCA |  |
|  |  | EU597619 | VIC |  | F: CTCTCATCCTTTGCAGCTGA  R: TGGGATGCACACTAATCTGC |  |
|  |  | AJ308769 | PET |  | F: TCCATCGTTTACGATTTGTC  R: GTCATCTATTTGTGAGCTTGG |  |
|  |  | EU597601 | NED |  | F: GCATCTTGATTCCCCTTCTC  R: GAATAGAGCGAGGCGTGTAT |  |
| D |  | EU597603 | FAM |  | F: TAAAGTGGATGCGTCTCCCA  R: GGATAAGCAAGGAGCTGCAA |  |
|  |  | EU597615 | VIC |  | F: GAGAGGATCATCGTGATCTTCG  R: CCGTCGTTATCTCCTATAAGCC |  |
|  |  | EU597627 | NED |  | F: ATGGACAGGAGTTGATGGTACT  R: CACTCATTTTGCCAATCTACC |  |
|  |  | AJ308776 | PET |  | F: TCTCCCTCTCCCTCTCTCT  R: GCGTTTGGTGGAGATGATA |  |
| E |  | EU597612 | NED |  | F: TGGTTGTGCTTACCCTACTAGG  R: TTGCAAACTTCTCCCGCTAG |  |
|  |  | EU597618 | VIC |  | F: TTGCTTGTCTTAGGTAGCCTG  R: CTAGAAGTGCCAAATGTGAGG |  |
| F |  | AJ250258 | FAM |  | F: AACTCTCCATTCCCGCATTC  R: CTGGGTTTTCTGTGTTCTCG |  |
|  |  | AJ308825 | PET |  | F: TTCTGGTTTCAACTCCATTT  R: ATAAACCCAAAAAGACCACA |  |
|  |  | EU597622 | VIC |  | F: AAGTGCCAAATGTGAGGCGT  R: AGAAAACACCATCACTCGGT |  |
| 1 GenBank Accession number (nucleotide record accessible via http://www.ncbi.nlm.nih.gov/nuccore/)  2 DS-33 Applied Biosystems® Standard Dye Set for Genotyping Applications | | | | | | |
